# Correlation Between the Orientation of the Nasopalatine Canal and the Upper Central Incisors: A Comparative Radiological Study Using Cbct

**DOI:** 10.3390/bioengineering12070719

**Published:** 2025-06-30

**Authors:** Alessia Lasaracina, Roberto Luongo, Sergio Aliota, Alessia Acquaviva, Calogero Bugea, Mariano Lauriola, Erda Qorri, Antonio Scarano, Sang-Choon Cho

**Affiliations:** 1 Private Practice, Via Niccolò Paganini 5, 70017 Putignano, BA, Italy; alessia.lasaracina99@gmail.com; 2 Arthur Ashman Department Perio/Implant Dentistry, College of Dentistry, New York University, New York, NY 10010, USA; 3 Private Practice, Via Firenze 16, 71019 Vieste, FG, Italy; saliota@hotmail.com; 4 Private Practice, Piazza Garibaldi 61, 70021 Acquaviva delle Fonti, BA, Italy; a.acquaviva@miulli.it; 5 Private Practice, Lungomare G. Galilei 133, 73014 Gallipoli, LE, Italy; calogerobugea@yahoo.it; 6 Lauriola Mariano Private Practice DDS, Via Scalette 41, 71037 Monte Sant’Angelo, FG, Italy; mariano.lauriola@gmail.com; 7 Department of Dentistry, Faculty of Medical Sciences, Albanian University, Tirana 1001, Albania; e.qorri@albanianuniversity.edu; 8 Department of Medical, Oral and Biotechnological Sciences, University of Chieti-Pescara, 66100 Chieti, CH, Italy; ascarano@unich.it; 9 Periodontology and Implant Dentistry, Ashman Department Perio/Implant Dentistry, College of Dentistry, New York University, New York, NY 10010, USA; scc2@nyu.edu

**Keywords:** implant placement, anatomical study, CBCT, incisive foramen

## Abstract

During implant placement in the upper anterior region, the nasopalatine canal (NPC) is a frequently encountered anatomical structure. It connects the nasal and oral cavities and contains critical blood vessels and nerves. Despite its clinical relevance, no study has yet assessed the orientation of the NPC in relation to the upper central incisors to aid in optimal implant positioning. This study investigated the parallelism between the NPC and the upper central incisors (1.1 and 2.1) in both the mesiodistal and buccopalatal directions. Data were collected from 226 subjects, and statistical analyses included Pearson’s correlation test, a one-sample *t*-test, and scatter plot analysis. The mean mesiodistal inclinations of the NPC, 1.1, and 2.1 were 87.54° ± 3.20, 86.55° ± 3.97, and 86.50° ± 3.63, respectively, while their buccopalatal inclinations measured 67.92° ± 6.89, 67.02° ± 6.88, and 67.23° ± 7.76, respectively. These findings indicate a strong correlation between the buccopalatal and mesiodistal inclinations of the NPC and the upper central incisors, with no significant differences observed. These results align with the existing literature on the anatomical variability and clinical significance of the NPC. This correlation suggests that evaluating the spatial relationship between the NPC and adjacent teeth could enhance implant surgery planning, leading to improved clinical outcomes and minimizing complications such as hemorrhage or paresthesia.

## 1. Introduction

The nasopalatine canal (NPC) is an anatomical structure located in the anterior maxillary region, first described by Stenson in 1683 [1]. It is positioned posterior to the maxillary central incisors and opens into the oral cavity near the incisive papilla, at the incisive foramen (IF) [2,3]. The NPC features two nasal openings, known as Stenson’s foramina (SF), located bilaterally on the floor of the nasal cavity adjacent to the nasal septum. Within the canal, several anatomical structures are housed, including the nasopalatine artery and nerve, small salivary glands, adipose tissue, and connective tissue [4]. The nasopalatine nerve, after merging with the greater palatine nerve, provides sensory innervation to the anterior portion of the hard palate and the lower third of the nasal septum [5,6].

**Figure 1:**
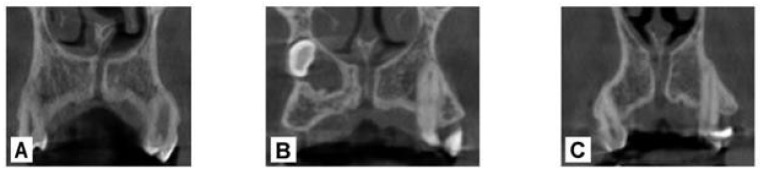
Anatomy of the nasopalatine canal in the coronal plane. (**A**) single canal, (**B**) double canal, (**C**) Y-shaped.

Several studies have reported morphological variations in the NPC in both the coronal and sagittal planes. In the coronal plane, Bornstein et al. classified the NPC into three distinct types: Type ‘a’, single canal; Type ‘b’, double canal; and Type ‘c’, Y-shaped, characterized by a single canal in the coronal portion and bifurcation in the apical portion [7] (Figure 1).

**Figure 2:**
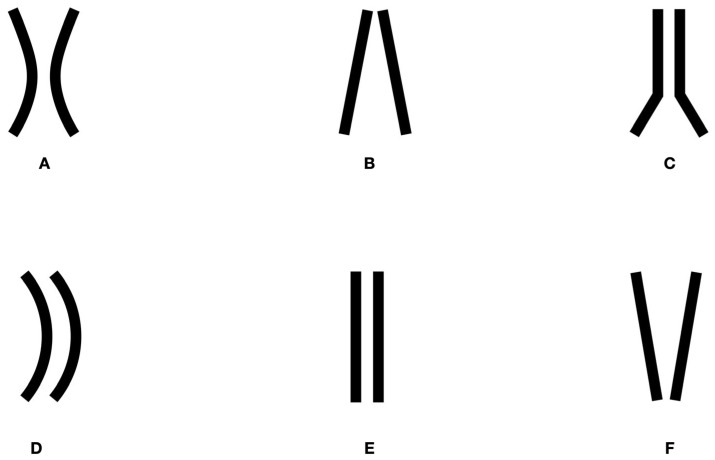
Classification of the nasopalatine canal in the sagittal plane. (**A**) Hourglass-shaped, (**B**) Conical, (**C**) Funnel-shaped, (**D**) Banana-shaped, (**E**) Cylindrical, (**F**) Inverted cone-shaped.

In the sagittal plane, the NPC has been classified into distinct morphological types based on its shape and structural variations [8,9]: hourglass-shaped (A), characterized by a central constriction where the canal walls narrow; conical (B), defined by walls diverging in an apico-coronal direction; funnel-shaped (C), exhibiting a wider coronal portion narrowing toward the apex; banana-shaped (D), displaying a curvature at the center of the canal; cylindrical (E), featuring parallel buccal and palatal walls; inverted cone-shaped (F), with walls converging in an apico-coronal direction [8,9] (Figure 2).

Given the sensitivity of the NPC, direct contact during surgical procedures may result in sensory alterations affecting the hard palate and portions of the nasal cavity. Additionally, if severed, the NPC poses a risk of intraoperative hemorrhage, emphasizing the need for precise pre-surgical diagnostic planning.

Cone-beam computed tomography (CBCT) is a crucial tool for pre-surgical planning, offering volumetric reconstructions of craniofacial structures similar to conventional multislice computed tomography (CT) but with shorter acquisition times, lower radiation doses, and reduced costs [10]. By providing three-dimensional (3D) imaging, CBCT overcomes the limitations of traditional two-dimensional (2D) methods [11]. This multidimensional assessment enhances surgical precision, reduces the risk of implant mispositioning, and minimizes intraoperative complications, improving patient safety [7].

According to Cawood and Howell’s classification of maxillary and mandibular bone resorption, basal bone resorbs last. This characteristic makes the NPC a crucial anatomical landmark in edentulous patients with advanced bone resorption, as it remains detectable even in cases of severe atrophy [12] and can serve as a reliable reference point for implant positioning in the upper jaw. Supporting this, a study demonstrated successful rehabilitation of severely atrophic patients using the NPC as an implant site [13].

In edentulous patients, where anatomical landmarks such as the central incisors are missing, implant placement often begins in the anterior maxillary region, near the midline. In this context, the nasopalatine canal serves as a stable and clearly identifiable reference on CBCT scans. Proper angulation in this area is critical, as the first two implants, placed parallel to each other and to the NPC, can guide the orientation of subsequent implants. This helps maintain surgical accuracy, reduces the risk of anatomical complications, and ensures parallelism, which is especially important in the atrophic maxilla. Maintaining consistent angulation facilitates prosthetically driven rehabilitation and helps avoid complications such as misfitting of metal frameworks or esthetic issues.

Given the clinical significance of the NPC in the maxilla, this study investigates the correlation between the NPC and adjacent anatomical structures, particularly the upper central incisors. Special attention was given to the parallelism between the NPC and upper central incisors, as this factor may play a key role in implant planning within the anterior maxillary region. Proper implant alignment in this area is essential to minimize the risk of neurovascular injuries.

To achieve this, this study employed CBCT to analyze the mesiodistal and buccopalatal inclinations of the upper central incisors relative to the NPC, providing valuable insights for improving implant placement precision and surgical outcomes.

## 2. Materials and Methods

This study analyzed a retrospectively selected sample of 226 patients (107 males, 119 females; aged 18 to 88 years; mean = 51, SD = 14), chosen from a CBCT scan database obtained for treatment planning at a private dental clinic (Istituto Stomatologico Mediterraneo, Bari, Italy).

The inclusion criteria required the presence of intact upper central incisors (1.1 and 2.1), complete visibility of the nasopalatine canal (NPC) in CBCT scans, and the absence of anatomical anomalies or pathologies affecting the examined structures.

Patients were excluded if they presented with severely compromised incisors, defined as teeth with fractures extending below the cemento-enamel junction, extensive carious lesions causing structural loss, root resorption visible on CBCT, or prosthetic restorations and large fillings that could interfere with the dental angular measurements. Patients with stage 3 or 4 periodontal disease, according to the current classification of EFP, were excluded by this study.

A power analysis indicated that at least 207 patients would be needed to detect a small to moderate correlation (r = 0.20) with a power of 90% and a significance level of 0.05. The final sample of 226 patients was therefore considered sufficient to ensure statistical significance.

This selection was based on the need to analyze anatomical relationships in a context where relevant structures were clearly identifiable and unaffected by bone loss.

Unlike the alveolar bone, which undergoes remodeling due to edentulism or periodontal disease, the NPC is located within basal bone, a region that remains structurally stable over time. Its orientation is therefore not influenced by the presence or absence of teeth, making it a reliable anatomical reference even in edentulous patients. Nevertheless, only patients with intact anterior teeth were included to maintain methodological consistency.

For each patient, high-resolution CBCT images were acquired and exported in DICOM format, allowing precise three-dimensional visualization of the examined anatomical structures.

All CBCT scans were acquired following a standardized clinical protocol using consistent exposure settings across all patients. Scans were performed with a voxel size of approximately 0.2 mm, tube voltage around 90 kV, and current between 8 and 10 mA.

The mesiodistal inclination (MDI) and buccopalatal inclination (VPI) of the upper central incisors (1.1 and 2.1) and the NPC were measured using Ez3Di image processing software, version 5.5.2, on sagittal and coronal sections.

**Figure 3:**
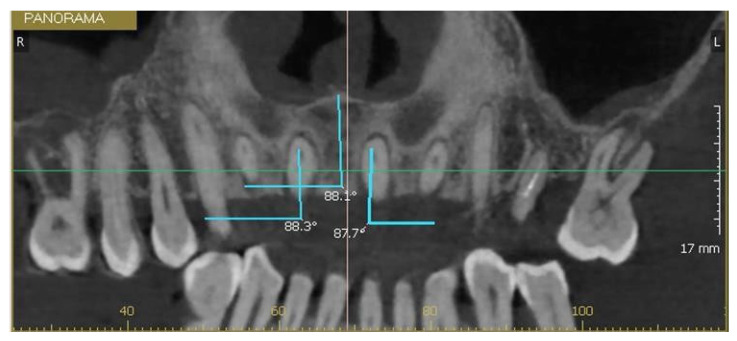
Mesiodistal inclination of tooth 1.1, the canal, and tooth 2.1.

**Figure 4:**
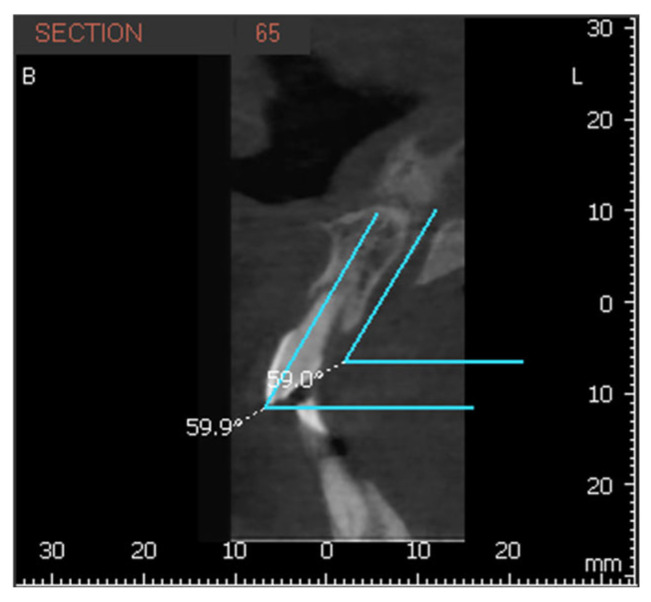
Buccopalatal inclination of tooth 1.1 and the nasopalatine canal.

**Figure 5:**
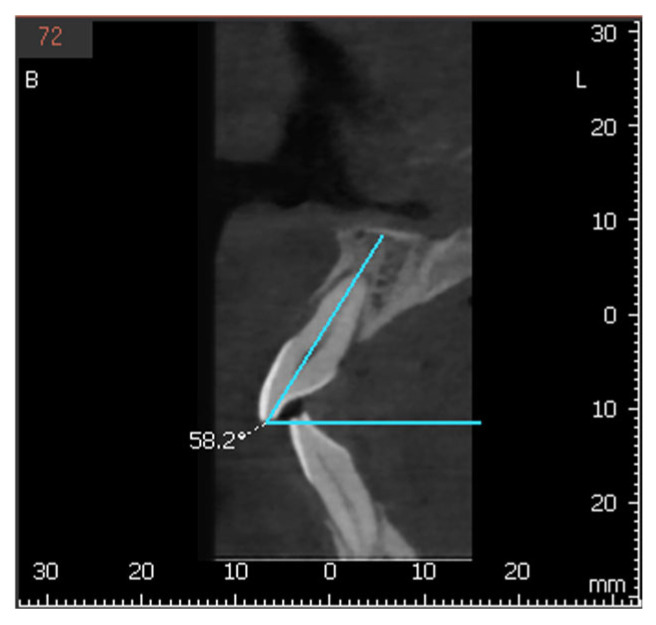
Buccopalatal inclination of tooth 2.1.

The longitudinal axis of each incisor was defined as the line connecting the incisal edge with the root apex, while the axis of the NPC was defined by marking a line between the nasopalatine foramen, superiorly, to the incisive foramen inferiorly. The MDI of 1.1 and 2.1 was calculated as the angle between their longitudinal axis and a transverse reference plane passing through the midline of the maxilla. The same measurement was applied to the NPC relative to this plane (Figure 3). The VPI of 1.1 and 2.1 was determined as the angle between their longitudinal axis and the horizontal plane, with the NPC measured in the same manner (Figure 4 and Figure 5).

For each subject and for each data group, the mean and standard deviation were calculated. Additionally, the differences between the mesiodistal inclination (MDI) and buccopalatal inclination (VPI) of each upper central incisor (1.1 and 2.1) and the corresponding NPC inclination were analyzed.

### 2.1. Intraclass Correlation Coefficient

To ensure accuracy and reproducibility, measurements were performed by two independent observers. Inter-observer reliability was assessed on a subset of 50 CBCT scans using the intraclass correlation coefficient (ICC), with values ranging from 0.91 to 0.95, indicating excellent agreement. To further reduce subjective bias during 3D reconstruction, both observers followed a standardized orientation protocol with consistent landmarks and software settings. High inter-rater consistency (ICC = 0.93) confirmed the reproducibility of the image alignment process.

### 2.2. Pearson Correlation Test

To assess the relationship between the inclinations of the upper central incisors and the NPC, the Pearson correlation coefficient was calculated. This analysis was conducted separately for both mesiodistal (MDI) and buccopalatal (VPI) inclinations, comparing the values of each incisor (1.1 and 2.1) with the corresponding NPC inclination.

### 2.3. One-Sample T-Test

To determine whether the differences between the mean inclinations of the upper central incisors and the NPC were significantly different from zero, a one-sample *t*-test was performed. This analysis was conducted separately for the mesiodistal (MDI) and buccopalatal (VPI) inclination differences.

The null hypothesis assumed that the mean differences were equal to zero, indicating a perfect parallelism between the incisors and the nasopalatine canal.

### 2.4. Graphical Analysis

Scatter plots were created to visualize the relationship between the inclinations of the incisors and those of the NPC. Separate graphs were created for mesiodistal (MDI) and buccopalatal (VPI) inclinations, with incisor inclination on the X-axis and NPC inclination on the Y-axis. Each plot included a linear trendline along with the coefficient of determination (R^2^) to illustrate the strength of the correlation.

### 2.5. Statistical Analysis

Statistical analyses were conducted using Microsoft Excel and additional data processing tools. A significance level (α) of 0.05 was applied to all tests.

For each Pearson correlation coefficient, a 95% confidence interval was also calculated using Fisher’s z-transformation.

Considering the presence of multiple comparisons, the Bonferroni correction was applied to adjust the significance threshold. For the four Pearson correlation tests performed, the corrected alpha level was set at 0.0125 (0.05/4).

Descriptive statistics, including mean, standard deviation, and sample size (n), were used to summarize the collected measurements.

## 3. Results

The MDI and VPI of the upper central incisors and the NPC were found to be 86.55° ± 3.97 and 67.02° ± 6.88 for 1.1, 86.50° ± 3.63 and 67.23° ± 7.76 for 2.1, and 87.54° ± 3.20 and 67.92° ± 6.89 for the NPC, respectively (Table 1).

The correspondence between these values was verified by calculating Pearson’s correlation coefficient.

The Pearson correlation coefficient for the buccopalatal inclination of tooth 1.1 relative to the NPC was 0.91 (95% CI: 0.88–0.93), *p*-value < 0.001, while for tooth 2.1, it was 0.81 (95% CI: 0.76–0.85), *p*-value < 0.001.

Regarding the mesiodistal inclination, the correlation coefficient was 0.71 (95% CI: 0.64–0.77), *p*-value < 0.001, for tooth 1.1 and 0.72 (95% CI: 0.65–0.78), *p*-value < 0.001, for tooth 2.1. In all cases, the correlation results reached statistical significance.

These findings indicate a stronger correlation in the buccopalatal direction for both incisors, while the mesiodistal inclination exhibits a weaker, yet still significant, relationship with the NPC (Table 2).

The *t*-test results revealed the following mean differences, t-values, and critical t-values (1.97) for the buccopalatal (VPI) and mesiodistal (MDI) inclinations:

- VPI of 1.1: mean difference: −0.90° | t-value: −4.71;
- VPI of 2.1: mean difference: −0.68° | t-value: −2.23;
- MDI of 1.1: mean difference: −0.98° | t-value: −5.22;
- MDI of 2.1: mean difference: −1.08° | t-value: −3.42.

In all cases, the calculated t-value was lower than the critical t-value (1.97), indicating that the differences between the inclinations of the upper central incisors and the nasopalatine canal (NPC) were not statistically significant.

These findings suggest that the upper central incisors are sufficiently parallel to the NPC in both the mesiodistal and buccopalatal directions, reinforcing the anatomical alignment between these structures.

The scatter plots illustrate the relationship between the inclinations of the upper central incisors and the NPC in both mesiodistal and buccopalatal directions.

**Figure 6:**
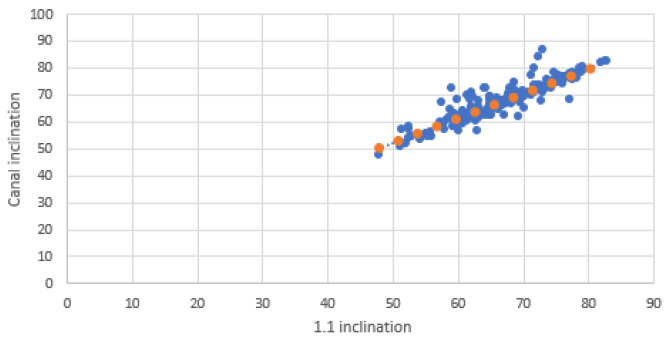
Buccolingual relationship between 1.1 and NPC. Blue dots represent individual data points; orange dots indicate values along the fitted trendline.

**Figure 7:**
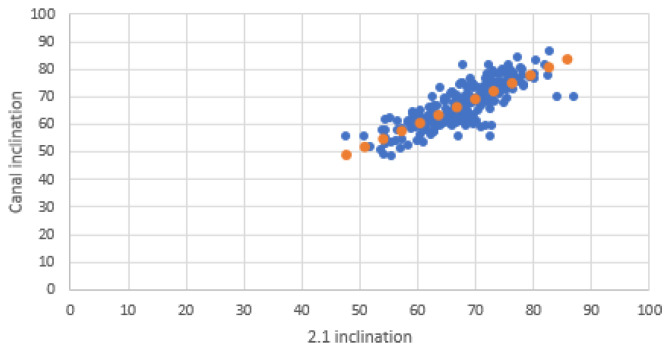
Buccolingual relationship between 2.1 and NPC. Blue dots represent individual data points; orange dots indicate values along the fitted trendline.

In the buccopalatal direction scatter plot, the inclinations of 1.1 and 2.1 exhibit strong alignment with those of the NPC, confirming the high correlation observed in Pearson’s analysis (Figure 6 and Figure 7).

**Figure 8:**
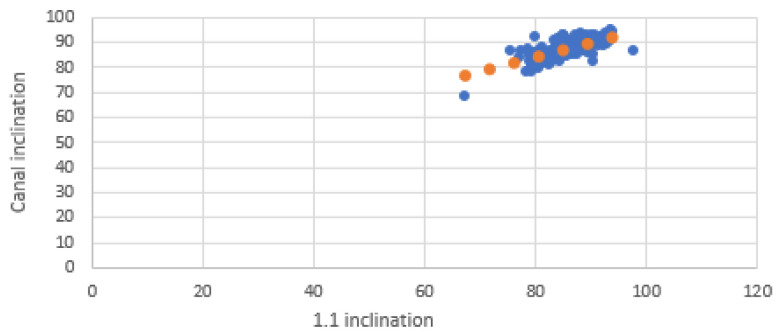
Mesiodistal relationship between 1.1 and NPC. Blue dots represent individual data points; orange dots indicate values along the fitted trendline.

**Figure 9:**
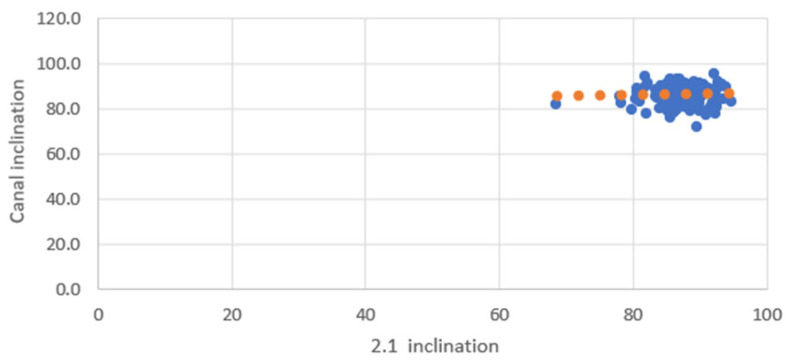
Mesiodistal relationship between 2.1 and NPC. Blue dots represent individual data points; orange dots indicate values along the fitted trendline.

In the mesiodistal direction scatter plot, the data points exhibit greater dispersion, reflecting the lower correlation values observed in Pearson’s analysis. However, despite this variability, the tendency toward parallelism between the upper central incisors (1.1 and 2.1) and the NPC remains evident (Figure 8 and Figure 9).

## 4. Discussion

The aim of this study was to evaluate the parallelism between the upper central incisors and the nasopalatine canal by measuring their respective mesiodistal and buccopalatal inclinations. The results revealed a strong positive correlation in the buccopalatal direction, with Pearson correlation coefficients of 0.91 for tooth 1.1 and 0.81 for tooth 2.1. These findings suggest that the buccopalatal inclinations of the upper central incisors and the NPC exhibit a strong tendency toward parallelism, which is particularly important for implant surgical planning. Proper alignment of these anatomical structures is crucial to minimizing the risk of neural and vascular complications.

In the mesiodistal direction, the correlation was moderate (0.71 for 1.1 and 0.72 for 2.1), indicating a general tendency toward parallelism but with greater variability compared to the buccopalatal inclination. However, these differences were not statistically significant.

The NPC has been widely studied by various authors who have also reported its clinical significance. In particular, some studies have explored the feasibility of severing the NPC’s neurovascular bundle, filling the canal with grafting material to facilitate guided bone regeneration (GBR), and subsequently using it as an implant site [14,15]. However, Urban et al. presented a series of clinical cases in which they opted to lateralize the neurovascular bundle instead of severing it, thus aiming at preserving the blood supply to the palatal mucosa, thereby reducing the risk of necrosis and membrane exposure [16].

Lateralization of the neurovascular bundle is a more conservative approach compared to its resection, as it minimizes the risk of bleeding and sensory disturbances while preserving palatal vascularization, which is an important factor in reducing flap necrosis and membrane exposure during GBR procedures [14,15,16].

The average length of the NPC has been documented by various authors, with studies identifying significant sex-related differences. Specifically, the mean NPC length was reported as 11.46 mm in men and 9.37 mm in women [17,18]. Additionally, another study found gender differences in canal diameter, with a mean measurement of 3.5 ± 0.81 mm in males and 2.9 ± 0.69 mm in females [19].

Regarding NPC morphology, Etoz and Sisman reported that 38.78% of NPCs exhibited an hourglass shape, making it the most common form, followed by funnel-shaped canals, which accounted for 27.35% of cases [20]. Less frequent variations included conical (9.18%) and cylindrical (8.25%) shapes. In contrast, Milanovic et al. [21], Lake et al. [22], and Linjawi et al. [23] identified the funnel shape as the most prevalent NPC morphology.

The position and inclination of the NPC are critical in preventing neurosensory complications such as paresthesia of the hard palate and nasal cavity floor, as well as intraoperative hemorrhage.

Even minor angular deviations can be a potential neurological and vascular risk during implant placement in the anterior maxilla. Recent studies on guided implant surgery have reported mean angular deviations ranging from 2.5° to 6°, and in some cases exceeding 8°, particularly in the anterior maxilla [24,25]. These findings suggest that a ±5° deviation threshold represents a clinically relevant safety margin. This article applies the ±5° deviation threshold criteria, showing that implant placement aligned with incisor inclination remains well within a safe range.

The NPC houses the nasopalatine nerve and artery, which provide sensory innervation and vascularization to the hard palate mucosa and nasal cavity floor. During implant placement, trauma or accidental contact with the nasopalatine nerve may lead to sensory disturbances, while damage to the nasopalatine artery could result in severe intraoperative bleeding. One study assessed the neurosensory function of the nasopalatine nerve in patients undergoing implant surgery after guided bone regeneration, with nerve lateralization in the anterior maxilla. Sensory function was evaluated through clinical examinations and patient questionnaires. While no patients reported pain or discomfort, 30% exhibited mild palatal sensory alterations, suggesting that although nerve lateralization is a predictable and safe technique, it still carries a risk of sensory impairment [16].

Since the NPC is located within the basal bone, it undergoes minimal changes despite the degree of bone ridge resorption. A thorough pre-implant assessment of the NPC is crucial to minimizing neurovascular complications and ensuring that implants in the 1.1 and 2.1 regions align with the parallelism of the NPC. Our findings indicate that following the mesiodistal inclination (MDI) of the NPC is essential to prevent damage to its neurovascular bundle during implant placement in this region.

The stronger buccopalatal correlation compared to the mesiodistal correlation may translate into important surgical implications, emphasizing the need to carefully assess mesiodistal inclination during implant planning. The greater variability observed in this direction suggests that heightened attention is required to prevent misalignment when preparing the implant site.

In general, implants should be placed using prosthetically guided techniques to prevent esthetic complications resulting from positioning and parallelism errors, especially when placing multiple implants. Although the use of a surgical guide can help mitigate these surgical challenges, it may be less precise in cases of severe atrophy. In these situations, the NPC could serve as an important anatomical landmark for accurate implant placement, both in esthetic areas and in multiple implant rehabilitations.

While numerous studies have examined the morphology, length, and anatomical variations in the nasopalatine canal (NPC), this study is, to our knowledge, the first to specifically evaluate its parallelism with the upper central incisors. This distinction makes our findings particularly significant, offering a new perspective on the anatomical relationship between these structures. The absence of comparable studies presents both a limitation and an opportunity. While it prevents direct comparison with previous research, it also highlights the need for further studies. Future research could explore additional variables, expand the sample size, or examine different population groups, further advancing our understanding of this anatomical correlation.

Despite the promising results, certain limitations must be acknowledged. Firstly, measurements were based on three-dimensional radiographic images, which, although highly accurate, may still be susceptible to errors related to resolution or image interpretation. Another potential limitation is the lack of stratification by age and gender. The study included both men and women across various age groups without distinction, potentially introducing variability in the data. Given that craniofacial anatomy evolves with age and differs between genders, this factor may have influenced the results. Future studies could enhance accuracy by conducting comparative analyses within homogeneous groups based on age or gender, allowing for a more precise evaluation of whether significant differences exist in the parallelism between the upper central incisors and the NPC.

## 5. Conclusions

This study demonstrates a significant parallelism between the upper central incisors and the nasopalatine canal, both in the buccopalatal and mesiodistal directions. Ensuring the proper inclination of implants in the upper central incisor region can help reduce the risk of canal penetration, thereby minimizing intraoperative complications and enhancing the safety and success of the procedure. These findings suggest that the NPC axis may serve as a reliable anatomical reference for implant angulation in clinical scenarios where natural dental landmarks are compromised. Future studies could further validate these findings clinically, exploring the potential of using the NPC as a reference point for implant parallelism in multiple rehabilitations and in cases where the upper central incisors are absent.

## Figures and Tables

**Table 1:** Mean values and standard deviations of mesiodistal inclination (MDI) and buccopalatal inclination (VPI) for upper central incisors (1.1 and 2.1) and the nasopalatine canal (NPC), measured in degrees.

Tooth/Canal	MDI	MDI Standard Deviation	VPI	VPI Standard Deviation
1.1	86.55°	3.97°	67.02°	6.88°
2.1	86.50°	3.63°	67.23°	7.76°
NPC	87.54°	3.20°	67.92°	6.89°

**Table 2:** Pearson correlation coefficients (r) for the mesiodistal (MDI) and buccopalatal (VPI) inclinations of teeth 1.1 and 2.1 in relation to the nasopalatine canal (NPC).

Tooth 1.1	Pearson Coefficient	Tooth 2.1	Pearson Coefficient
Buccopalatal	0.91	Buccopalatal	0.81
Mesiodistal	0.71	Mesiodistal	0.72

## Data Availability

The data presented in this study are available on request from the corresponding author due to privacy.
